# Seminal dysbiosis in idiopathic male infertility: toward a seminal dysbiosis–inflammatory/oxidative endophenotype–SDF axis

**DOI:** 10.3389/fendo.2026.1943471

**Published:** 2026-09-28

**Authors:** Yuan Gao, Xiaoqia Lv, Zixue Sun

**Affiliations:** 1 Henan University of Chinese Medicine, Zhengzhou, China; 2 Henan Province Hospital of Traditional Chinese Medicine (The Second Affiliated Hospital of Henan University of Chinese Medicine), Zhengzhou, China

**Keywords:** idiopathic male infertility, inflammatory/oxidative endophenotype, oxidative stress, seminal dysbiosis/seminal microbiome dysbiosis, sperm DNA fragmentation

## Abstract

Idiopathic male infertility (IMI) usually refers to abnormal semen parameters after known causes have been excluded. However, its clinical stratification and intervention evidence remain difficult to reconcile. Based on low biomass in semen, this review proposes an SD–IO–SDF axis linking seminal dysbiosis (SD), inflammatory/oxidative endophenotype (IO), and sperm DNA fragmentation (SDF) in subsets of IMI. It integrates three mechanisms of evidence strength: (i) inflammation–oxidative injury pathway (leukocyte-dependent); (ii) leukocyte-independent redox imbalance; and (iii) a metabolic–epigenetic route (SAM cycle, butyrate) as a testable hypothesis. Existing evidence suggests that the association between SD and SDF concentrates in specific IO activation states and IMI subtypes. IO stratification may reduce effect dilution across mixed populations and make the hypothesis more directly testable. For research implementation, we propose IO indicators grouped by measurement type (inflammatory: leukocytospermia and/or PMN-elastase; oxidative: sORP), with validation against SDF platforms as a subsequent step. This approach provides a falsifiable structure for IMI and provides a template for future trial designs based on SD×IO stratification.

## 1 Introduction

Globally, infertility affects approximately 8%–12% of couples, and male factors account for about 50% of cases (1). Routine evaluation of male infertility relies mainly on medical history, physical examination, and semen analysis, but these measures have limited value for explaining the underlying pathology (2). Idiopathic male infertility (IMI) is defined as abnormal semen parameters with no identified cause and accounts for about 30% of male infertility cases (3). In a substantial proportion of IMI patients, routine testing does not capture the underlying disease process (4). The 2024 AUA/ASRM guidelines also stress that stratification and advanced diagnostic workup ought to be guided by abnormal results of initial assessment (5). Mechanistically, oxidative stress reflects redox imbalance, while sperm DNA fragmentation reflects the downstream genomic damage it may cause. These remain the two most extensively studied mechanisms in this field (6, 7).

Interest has grown in whether dysbiosis of the seminal microbiome is associated with inflammatory or oxidative endophenotypes in some IMI patients. Across infertility cohorts, *Prevotella*/*Atopobium* enrichment often co-occurs with higher oxidative stress and sperm DNA damage, whereas *Lactobacillus* enrichment is associated with a more favorable redox state (8, 9). In normozoospermic infertile men compared with sperm donors, the abundance of specific genera has been associated with DNA fragmentation and chromatin integrity (10). Semen is a low-biomass sample, which limits inference from microbiome association studies (9, 11). The present analysis is restricted to IMI cases meeting the quality-control criteria defined below. It builds on Male Oxidative Stress Infertility (MOSI) by incorporating seminal dysbiosis, with inflammation/oxidative stress as the measurable intermediate layer (12). While SD, IO, and SDF show associations across cohorts, an integrated framework for stratified testing across these three dimensions is still lacking. This narrative review proposes the SD–IO–SDF axis for IMI, defines the operational boundaries of SD, IO, and SDF, and discusses stratified diagnosis and trial design accordingly.

## 2 Background and methods

### 2.1 Key concepts and scope

#### 2.1.1 Operational definition and subtypes of IMI

IMI here is defined strictly as abnormal semen parameters after known causes have been excluded. In contrast, unexplained infertility refers specifically to couples with normal semen analysis, no abnormalities in partner evaluation, and persistent failure to conceive; these terms should not be used interchangeably in the literature (13–15). Studies should report, at minimum, hormone levels, testicular ultrasound, and a complete semen analysis. Genetic and microbiological testing should follow guideline-based indications (13). Traditional subtype classifications, such as oligoasthenozoospermia, describe phenotype only and do not explain mechanism. Accordingly, this synthesis is restricted to idiopathic cases with ejaculated sperm available and abnormal semen parameters (**Table 1**).

**Table 1:** Inclusion and exclusion criteria and subtype definitions for IMI studies.

Subtype	WHO 2021 semen criterion (lower reference limit)	SD–IO–SDF axis notes (minimal)
Idiopathic oligozoospermia (iOligozoospermia)	Sperm concentration < 16 × 10^6^/mL	Report how sORP is normalized (concentration is the denominator).
Idiopathic asthenozoospermia (iAsthenozoospermia)	Progressive motility < 30%	Motility phenotype; IO/mitochondrial mechanisms are often discussed (16).
Idiopathic teratozoospermia (iTeratozoospermia)	Normal morphology < 4% (strict)	Direct SD–morphology evidence is limited; subtype often underreported in SD studies.
Idiopathic oligoasthenoteratozoospermia (iOAT)	Concentration, progressive motility, and morphology all below the above thresholds	Mixed phenotype; SD signals can be diluted without IO/SDF stratification.

WHO 2021 lower reference limits were used: 16 × 10^6^/mL, 30% progressive motility, and 4% normal forms (15).

#### 2.1.2 Definition of the seminal microbiome

The “seminal microbiome” refers to the microbial community in ejaculated semen and its genetic material. It can be assessed by culture-based methods, 16S rRNA amplicon sequencing, and shotgun metagenomics, but these methods differ in resolution and bias profiles (17, 18). Ejaculated semen is a mixed fluid from multiple compartments. The similarity between the semen and urine microbiomes suggests that the urethra contributes to semen samples, and approximately 30% of the seminal microbial community may originate from the urethral microbiome (17, 19). Seminal plasma biochemical markers can help interpret microbiome data (20). Testicular biopsy or epididymal samples more closely reflect proximal compartments, but these procedures are invasive and have limited clinical feasibility (21). Because semen is a low-biomass sample, quality-control and decontamination requirements are described in Section 2.2 (22). Culture-based methods have a much lower detection rate for anaerobes and fastidious organisms (18). Clinically diagnosed reproductive tract infection has pathogen-based criteria, whereas leukocytospermia is an inflammatory marker and is not equivalent to infection (23).

Here, “seminal microbiome” denotes community structures characterized by next-generation sequencing (NGS) data after decontamination. Microbial detection is not interpreted as infection. Immune or inflammatory mechanisms are used only in studies that examine the co-occurrence of microbial load and host inflammation; metabolic mechanisms are used only in studies with functional or metabolic evidence (24). As a result, cross-study comparisons in Section 3 are limited by heterogeneity in detection platforms.

#### 2.1.3 Hierarchy of outcome measures

This synthesis covers outcome measures at multiple levels. If they are mixed together, cross-sectional correlations may be overinterpreted as causal sequences.

L1: Live birth rate and clinical pregnancy rate. Improvements in surrogate endpoints do not necessarily translate into live birth benefits. Interventions that use changes in semen parameters as endpoints may have limited impact on live birth probability (25). The AUA/ASRM guidelines state that, for couples with assisted reproductive technology (ART) failure or two or more pregnancy losses, male karyotyping and sperm DNA fragmentation should be assessed (5).

L2: SDF and IO measures. SDF, a central readout in this axis, has been linked in systematic reviews and meta-analyses to reproductive outcomes such as pregnancy loss (26, 27). IO is treated here as a candidate intermediate phenotype.

L3: Routine semen parameters and semen microbiome measures. Clinical trials in male infertility often rely on semen analysis and other surrogate endpoints to assess intervention effects (13).

Interpretation rules: Associations between SD and IO should be reported only as association signals. Associations between IO and SDF are treated as provisional findings once the methodological requirements in Section 2.2 are met. Surrogate endpoints, including ART/ICSI (intracytoplasmic sperm injection) outcomes themselves, cannot fully rule out confounding from maternal factors. Their predictive value remains controversial (28–30). For this reason, an upper limit on evidence strength is applied to each outcome level throughout this synthesis.

### 2.2 Methodology and quality control

Semen is a low-biomass sample, and contamination and batch effects can substantially influence the results (9, 31). Any claim about SD ecotypes should first be assessed through decontamination procedures, and reports should include negative controls, batch information, and detection thresholds (31–33).

Contamination can occur during sample collection, DNA extraction, amplification and library preparation, and sequencing. When negative controls are unavailable, *de novo* identification tools such as Squeegee can serve as a supplement (34, 35). No single decontamination tool is universally optimal. The choice should depend on study design and downstream analytic goals; micRoclean, for example, offers two alternative approaches (36). Because these samples have mixed sources, source tracking is also needed to distinguish signals from urethral colonization, skin contamination, or transfer from a partner (37, 38). The QIIME2 platform lists FEAST and SourceTracker2 as optional source-tracking methods. These two methods differ in their statistical assumptions, and the choice mainly depends on the study’s prior assumptions (37, 39).

Relative abundance can generate spurious associations in correlation analyses. We therefore recommend reporting absolute quantification, such as qPCR or flow cytometry, and describing the quantification method in the Methods section (40–42).

Both the IO and SDF layers also face methodological constraints. The normalization approach and cutoff values for sORP vary substantially across studies. Samples with high sperm concentration, extreme pH, or combined severe oligozoospermia and necrozoospermia should be interpreted with particular caution (6). Applying cutoff values across studies usually requires local validation (43–48). When sample pH is ≥ 8, two measurements of the same sample can differ by more than 10 mV, which further limits cross-laboratory comparability of cutoff values (45). Indicators that can assess the redox state of semen/seminal plasma include reactive oxygen species (ROS), oxidation-reduction potential (ORP) —when measured in seminal plasma, referred to as sORP, total antioxidant capacity/FRAP, lipid peroxidation markers such as MDA, and 8-OHdG, a marker of oxidative base damage (6, 49).

For SDF, the required reporting items include the assay method and readout type (SCSA-derived DNA fragmentation index [DFI], TUNEL, SCD, or Comet, with the acidic or alkaline protocol specified), sample form (fresh or freeze-thawed), sample condition, liquefaction time, abstinence period, laboratory quality control, and the source of the cutoff. Recommended items include concurrent reporting of high DNA stainability (HDS) with SCSA to add information on chromatin maturation. Double-strand break (DSB) oriented readouts may also be included (14, 15, 50).

The STORMS minimum reporting set specifies sample-level items (abstinence period, collection method), experimental-level items (total bacterial load, negative controls, batch information), and analysis-level items (decontamination method, thresholds, source tracking). These requirements also apply to IO and SDF testing (51).

Another independent threat to inference is reverse causality. Observed microbial differences may reflect SD-associated IO or SDF abnormalities. They may also reflect reverse selection on the microbiota after changes in IO or the seminal niche. Cross-sectional data cannot distinguish between these two directions, which sets the upper limit on how these associations should be interpreted here.

These confounders were incompletely reported in most studies included in this review. Given these gaps, study designs should at least control or report host and stratification variables (**Table 2**).

**Table 2:** Key confounders in seminal microbiome studies (minimum reporting items).

Domain	Report (minimum)	Why
Timing, sex, partner	Abstinence (2–7 d); recent ejaculation/sex frequency; recent partner change/cohabitation; urination before collection	Rapid short-term shifts (9)
Drugs, supplements (past 1–3 months)	Antibiotics; probiotics or synbiotics; antioxidant supplements	Direct exposure effects (52–54)
Sample and lab pipeline	Specimen (whole semen, seminal plasma, sperm pellet); ejaculate fraction collected; any wash, swim-up, gradient; time and temperature to processing or freezing	Pipeline changes the signal (19, 22)
Host background	Smoking; alcohol; BMI or metabolic disease; recent fever or systemic inflammation; varicocele status (exclude from IMI; report grade if present)	Oxidative stress and inflammation confounding (9)
Semen milieu at sampling	Leukocytospermia; pH; viscosity or liquefaction time; seminal PMN-elastase	Niche and inflammation markers (55)
Seminal biochemistry	Fructose (seminal vesicle), zinc and citrate (prostate), NAG and GPC (epididymis); report if any marker is below reference	Defines substrate niche for microbiota (20, 56)

For timing, the long abstinence of 2 days (vs. 2 hours) raises bacterial load (57); abstinence >7 d raises DFI, lowers motility (58). For host background, record febrile illness (≤90 d) including SARS-CoV-2 infection; COVID-19 vaccination and post-vaccination fever (59, 60).

### 2.3 Search strategy, study selection, and scope

This narrative review is based on a focused literature search rather than a systematic review; no PRISMA process or formal study-selection count was performed. We searched PubMed/MEDLINE, Embase, and Web of Science Core Collection from inception to September 6, 2026, using the following term categories: (1) idiopathic male infertility, oligozoospermia, asthenozoospermia, teratozoospermia, oligoasthenoteratozoospermia; (2) seminal microbiome terms, including seminal microbiome, semen microbiota, 16S rRNA, shotgun metagenomics, dysbiosis, contamination, and low-biomass; (3) inflammatory and oxidative endophenotype markers; and (4) sperm DNA fragmentation and reproductive outcomes. Guideline documents from AUA/ASRM, EAU, and the WHO Semen Analysis Manual were also reviewed. We also snowballed the reference lists of the included reviews and key original studies to identify potentially missed methodological and mechanistic literature.

In total, this synthesis draws on 8 representative association studies (**Table 3**), 5 intervention studies (**Table 4**), and 9 guideline/recommendation documents. Studies were selected to represent the breadth of association signals and mechanistic diversity relevant to the SD–IO–SDF axis, not to achieve exhaustive coverage of all eligible literature. The existing seminal microbiome literature may be affected by publication bias. Negative or nonsignificant studies are less likely to be published. As a result, the positive associations summarized here may appear stronger than their true effect sizes.

**Table 3:** Representative research stratification.

Study	Study design (n)	Population tag	Platform (QC)	Key finding	Evidence
Lundy et al. (2021) (61)	25 idiopathic infertility + 12 fertile	Extended	16S+shotgun (QC not reported)	Prevotella ↓ sperm concentration; SAM-cycle shifted	SD-L3; Route C (L3)
García-Segura et al. (2022) (10)	42 normozoospermic infertility + 14 donors	Extended	16S (blanks reported)	Taxa associates with ORP and sperm DNA integrity	SD-L3; SD-SDF (L2)
Mowla et al. (2025) (22)	223 mixed MFI/UI/RPL + fertile	Extended	16S+qPCR (blanks reported)	Microbiota and load show no consistent ROS or SDF link	SD-IO; SD-SDF (L2)
He et al. (2024) (62)	47 high DFI + 40 low DFI	Extended	16S+metabolomics (blanks+decontam)	L. iners and butyrate pathways enriched in high DFI	SD-SDF (L2)
Lu et al. (2025) (63)	40 idiopathic oligoasthenozoospermia + 30 fertile	IMI-core	16S+metabolomics (QC not reported)	Dialister inversely correlates with L-acetylcarnitine	SD-L3 (L3)
Panner Selvam et al. (2022) (64)	Meta-analysis	Meta-analysis	N/A (pooled)	Higher ORP correlates with higher SDF	IO-SDF (L2)
McQueen et al. (2019) (27)	Meta-analysis	Meta-analysis	N/A (pooled)	Higher SDF associated with RPL	SDF-L1 outcome (L1/L2)
Szabó et al. (2023) (65)	Meta-analysis	Meta-analysis	N/A (pooled)	Multiple host/clinical risk factors are associated with higher SDF	Host factors-SDF (L2)

Extended populations were included solely for hypothesis generation (Section 2.3); their findings are not used to support the core SD–IO–SDF evidence chain for IMI. Population tag: IMI-core = meets Section 2.1.1 criteria; Extended = non-IMI cohorts, RPL, or mixed phenotype cohorts; Meta-analysis = systematic reviews or meta-analyses. Evidence labels indicate outcome level (L1–L3, Section 2.1.3); all studies in this table are classified as preliminary evidence due to design and assay heterogeneity. Platform (QC) reflects sequencing method and decontamination status only.

**Table 4:** Representative intervention studies in idiopathic male infertility and related populations.

Intervention	Population scope	Key endpoint findings
Synbiotic + antioxidant (SperiGen); double-blind RCT (iOAT) (66)	IMI-core	Progressive motility ↑; DFI ↓; pregnancy/live birth not reported
Probiotic–prebiotic (Flortec); RCT (iOAT) (67)	IMI-core	Semen parameters ↑; IO/OS not measured; fathered a child (corollary) ↑; SDF (SCD) ↓
Doxycycline (low-level leukocytospermia) (68)	Extended	Semen parameters ≈ unchanged; leukocytospermia resolution ↑; natural pregnancy ↑; SDF not reported
SUMMER RCT (69)	Extended	ongoing pregnancy ≈ unchanged; SDF ≈ unchanged; sperm vitality ↓
MOXI RCT (70)	Extended	Semen parameters ≈ unchanged; DFI ≈ unchanged; live birth ≈ unchanged

IMI-core = meets Section 2.1.1 criteria; Extended = non-IMI cohorts. Trials did not measure the seminal microbiome or select treatment by SD ecotype. OS, oxidative stress; SDF, sperm DNA fragmentation; DFI, DNA fragmentation index; SCD, sperm chromatin dispersion; RCT, randomized controlled trial; iOAT, idiopathic oligoasthenoteratozoospermia. ↑/↓, statistically significant increase/decrease; ≈, no statistically significant change vs. comparator. SperiGen and Flortec are the commercial synbiotic/probiotic-prebiotic products evaluated in the respective trials.

Population: IMI with abnormal semen parameters (Section 2.1.1). Normozoospermic infertility, RPL, and HDFI (high DFI) cohorts were used for hypothesis generation only; infection-positive and varicocele cohorts were excluded. Idiopathic non-obstructive azoospermia was also excluded, as ejaculated sperm are absent and testicular microbiome data are not directly comparable to ejaculated seminal microbiome findings.

Technical criteria: NGS-based microbiome studies of ejaculated semen/seminal plasma, or clinical studies with IO/SDF co-measurement. Methodologically inadequate studies are classified as preliminary evidence (Section 3.3).

## 3 Evidence synthesis

### 3.1 Seminal microbiome (not restricted to a specific population)

Microbiome findings below are interpreted under the low-biomass quality-control and decontamination requirements described in Section 2.2. When studies do not meet the minimum threshold, we report only the direction of association. Existing NGS studies suggest that the seminal microbiome often forms genus-dominant profiles, most commonly *Lactobacillus*-dominant or *Prevotella*-dominant (9). The former is often associated with better semen parameters, the latter to poorer sperm quality. However, most findings remain at the genus level, and standard 16S regions usually do not provide species-level resolution (9). At present, the more consistent signals come from associations between specific genera and semen parameters, ROS, and DNA fragmentation, rather than from global microbiome disruption itself (22, 71, 72).

Functionally, the SAM cycle (S-adenosyl-L-methionine cycle) shows some of the clearest differences between fertile and infertile men, a pattern supported by subgroup analyses of semen and urine (61). In mixed infertility cohorts, seminal α-diversity is often higher; *Prevotella* abundance is negatively correlated with sperm concentration; while Pseudomonas abundance is positively correlated with total motile sperm count (61). Community profiles dominated by *Prevotella* and *Porphyromonas* are associated with poorer ART outcomes (73). In a small set of couple-level studies (n = 9), within-couple genital microbiomes were consistently more alike than between-couple profiles, even after controlling for abstinence and hygiene. Shared genera were frequently *Lactobacillus*; *Gardnerella* and *Prevotella* were also commonly detected in both partners (9). At present, however, no single biomarker can reliably distinguish fertile from infertile men (74).

### 3.2 Evidence synthesis: associations between SD and IO/SDF

#### 3.2.1 Idiopathic infertility vs fertile controls

Direct IMI evidence is sparse; extended populations were used for hypothesis generation (Section 2.3). Normozoospermic infertility studies (extended population) linked Brevundimonas and Flavobacterium to DFI and ORP (10). A phenotypically mixed cohort (n = 223; extended population per Section 2.3) found no stable association between microbiome and ROS or SDF (22), consistent with the prediction that pooling unstratified phenotypes dilutes detectable effects (24, 75, 76). Subtype-specific evidence is discussed in Sections 3.2.2–3.2.3.

#### 3.2.2 High DFI/redox-abnormality phenotype and microbiota-metabolite coupling

HDFI cohorts provide hypothesis-generating signals, with clearer DFI readouts and stronger outcome linkage (77). *Lactobacillus iners* is associated with high DFI. Butyrate is a candidate target metabolite, and its possible role is discussed in Section 4.4 (62).

Elevated proinflammatory cytokines in seminal plasma, such as IL-6 and TNF-α, often co-occur with reduced motility, increased DNA fragmentation, and granulocyte-derived oxidative stress. Across infertility cohorts, cross-sectional data are compatible with a “microbiota–inflammation/oxidative stress–sperm dysfunction” pattern (9). Across studies reviewed by Kuribayashi et al., leukocytospermia co-occurred with higher community diversity and enrichment of specific genera, such as *Streptococcus* (9). Semen parameters were also poorer. The leukocyte counting method and recent antibiotic exposure were reported inconsistently across studies (9, 19, 78). Leukocytospermia may also be driven by non-infectious factors such as oxidative stress or prolonged abstinence (79, 80). It does not always indicate urogenital infection; leukocyte infiltration linked to subclinical inflammation is one possible route to increased SDF, but not the only one (78). These two discrepancies partly reflect differences in detection methods and may also depend on whether the population was stratified by IO (6, 17, 81, 82). Functional evidence also points to a metabolic route independent of overt inflammation. The SAM-cycle changes noted in Section 3.1, the genus–ORP and genus–DSB associations in Section 3.2.1, and the butyrate signal above together suggest microbial metabolites as a possible mechanism (10, 83). These remain parallel association rather than established causal links. The need for longitudinal validation is discussed in Section 5.3.

The butyrate–*Lactobacillus iners* signal and the co-occurring inflammatory cytokine signal are both cross-sectional associations. This remains preliminary evidence (single study/no replication). This does not match the overall genus-level pattern in Section 3.1, suggesting that species-level resolution may be a key source of confounding. This inconsistency is discussed as a hypothesis in Section 4.4.

#### 3.2.3 Subtype-specific imbalance patterns in iAsthenozoospermia and iOAT

In iAsthenozoospermia, increased Lactobacillus abundance is not consistently associated with better motility (17). In iOAT, the pattern more often involves changes in mitochondrial energy-related pathways and the antioxidant system, and inflammation may not be the only intermediate phenotype (63, 84). Together with the butyrate signal observed in the HDFI population (Section 3.2.2), these observations suggest that the functional consequences of SD may be subtype dependent. Independent cohort validation is still needed.

### 3.3 Evidence assessment

The evidence presented here is classified as preliminary, because the relevant association studies rely on a single study, lack replication, or use highly heterogeneous reporting methods.

IO - SDF: A systematic review and meta-analysis reports a positive association between seminal oxidation–reduction potential (ORP) and SDF across clinical datasets (pooled r = 0.24; I² = 42.27%), but the evidence base is observational and heterogeneous in ORP normalization and SDF platforms/thresholds (64). Certainty therefore remains low and is classified as preliminary evidence.

SD - IO: Anaerobe-predominant communities, often involving *Prevotella* or *Atopobium*, together with elevated proinflammatory cytokines (*IL-6*, *IL-8*, *TNF-α*), are associated with poorer redox and DNA-integrity outcomes. This association is also classified as preliminary based on the evidence in Section 3.2.1 (9, 61). No independent replication in an IMI-core cohort is currently available.

SD - SDF: In men with idiopathic infertility and no overt inflammation, seminal dysbiosis may impair sperm function; however, its predictive value for SDF is unstable across mixed populations and requires reassessment following IO stratification, pending independent replication beyond the single available cohort (10).

## 4 Core framework: proposing and deconstructing the SD–IO–SDF axis

### 4.1 Deconstructing the SD, IO, and SDF

The evidence reviewed in Section 3 is predominantly cross-sectional. Accordingly, Pathways A and B and Route C (**Figure 1**) are presented as an ordered sequence for stratified testing rather than a proven causal chain; reverse causality cannot be excluded without the longitudinal designs discussed in Section 5.3. The feedback loop between IO and microbiome composition is described separately in Section 4.5.

**Figure 1:**
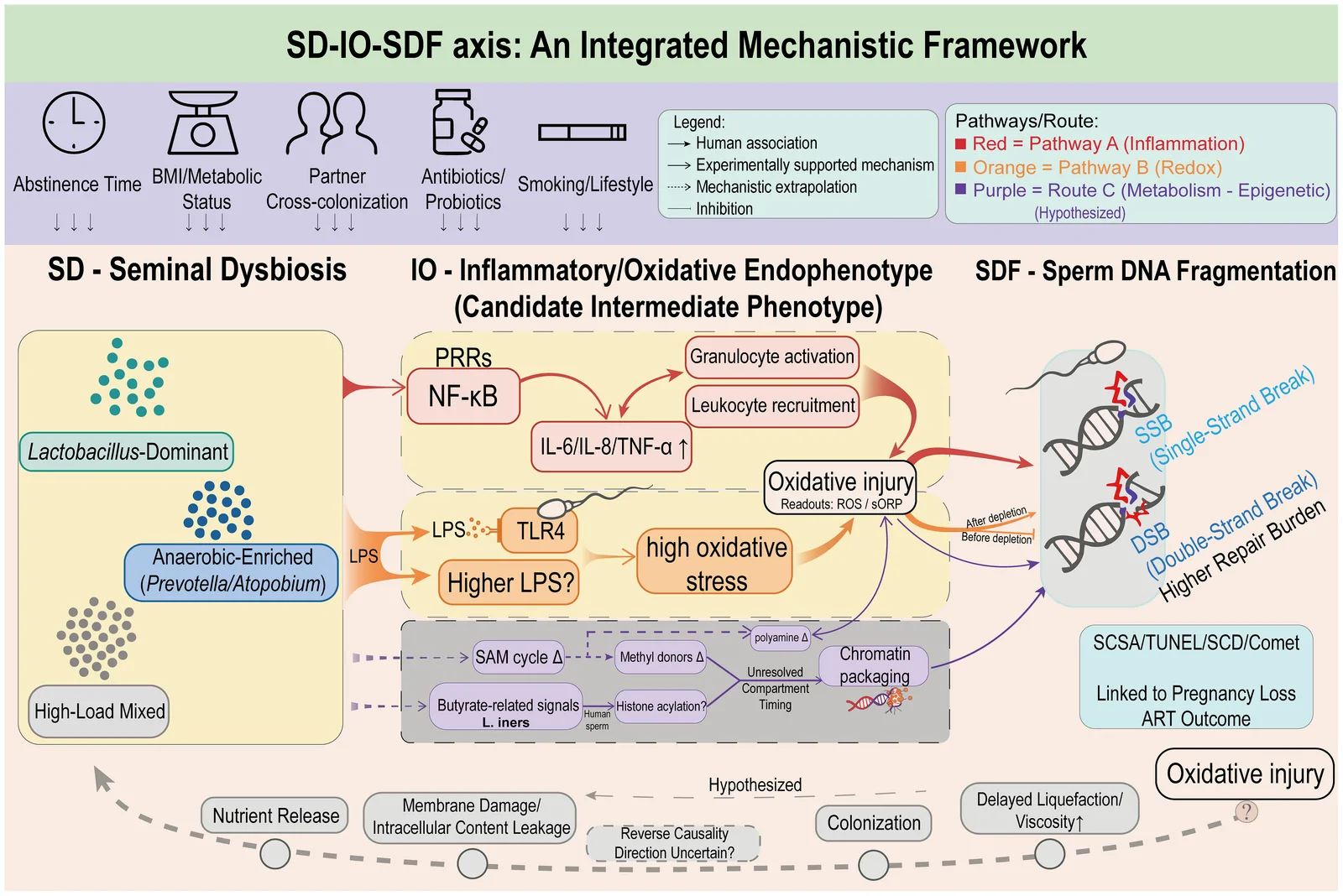
Mechanistic diagram of the SD–IO–SDF axis.

#### 4.1.1 SD: seminal dysbiosis

SD (seminal dysbiosis) refers to reproducible shifts in seminal microbial community structure identified under strict low-biomass quality control (61, 85). Here, SD is a candidate stratification variable for hypothesis testing. Relative abundance alone cannot explain the outcome. Its relationship with SDF should be assessed after IO stratification (86–88).

#### 4.1.2 IO: inflammatory/oxidative endophenotype

IO is an independently measurable candidate intermediate phenotype layer between SD and SDF. Leukocytospermia, PMN-elastase, and elevated cytokines serve as inflammatory readouts, while sORP can serve as an oxidative readout (12, 14, 89–93).

Leukocytospermia is the most accessible inflammatory marker in semen, but its specificity is limited. Seminal PMN-elastase can complement leukocyte counts; it is especially useful in samples in which leukocyte counts do not reach the threshold but granulocytes are already activated (94). These two measures serve as the initial operational indicators for the inflammatory component of IO (14, 91, 92). Cross-sectional studies suggest that leukocytospermia co-occurs with specific seminal microbiome patterns and is accompanied by lower relative abundance of *Lactobacillus* and poorer semen parameters (87, 90).

Seminal cytokine profiles provide a complementary IO readout. Elevated seminal *IL-6*, *IL-8*, and *TNF-α* have often been reported together with reduced sperm motility, increased DNA fragmentation, and higher ROS burden. Mechanistically, they may promote ROS production through immune cell activation and the NADPH oxidase pathway (9, 78, 93). In some samples, colonization or infection co-occurs with leukocytospermia, suggesting that bacteria may trigger leukocyte recruitment (55, 92). Conversely, inflammation may also alter the seminal niche and thereby affect microbiome stability and persistent colonization.

sORP, measured by MiOXSYS, is a practical oxidative stress readout for IO and is the marker used within the MOSI framework (12, 44–46). Given between-study differences in cutoff values and normalization methods (see Section 2.2), this review uses sORP only to determine whether IO is activated at the stratification stage (64, 89).

Stratifying by IO before testing the relationship between SD and SDF may reduce effect dilution in mixed populations (12, 22). Within the inflammatory component, leukocyte count, granulocyte activation state (PMN-elastase), and cytokine signaling (IL-6, IL-8, TNF-α) capture different readouts and are not interchangeable. Elevated sORP without leukocytospermia is often interpreted as noninflammatory oxidative imbalance. When neither inflammatory nor oxidative IO readouts are clearly abnormal, Route C (Section 4.4) remains a candidate mechanism (95).

#### 4.1.3 SDF: sperm DNA fragmentation

SDF quantifies nuclear DNA damage in sperm. The EAU guidelines recommend SDF assessment in settings such as recurrent pregnancy loss. Platform-specific readouts (SCSA-DFI, TUNEL%, SCD, Comet) are cited using their original labels throughout (14). The minimum reporting items are listed in Section 2.2.

The type of DNA break can provide clues about severity. Single-strand breaks (SSBs) and oxidative base damage are more often consistent with the ROS mechanism. DSBs carry a greater repair burden. In some reviews, they show a more consistent association with pregnancy loss, but this remains affected by detection strategies and population heterogeneity (96). The oocyte’s ability to repair paternal DNA damage can modify the clinical effect of SDF, and this capacity may vary with maternal age (97).

### 4.2 Mechanistic pathway A: inflammation- associated oxidative damage (leukocyte infiltration and granulocyte activation)

In subgroups with increased microbial load or consistently abnormal host IO readouts, SD may be associated with greater pathogen-associated molecular patterns (PAMPs) exposure. This can activate local innate immunity, shift seminal plasma toward a more inflammatory and oxidative state, and be associated with higher SDF (**Figure 1**) (22, 98, 99).

Pattern recognition receptor (PRR) responses in the reproductive tract epithelium and immune cells can activate pathways such as *NF-κB*. This may increase cytokine secretion and leukocyte recruitment (91). *IL-6* can be detected stably in semen even in the absence of overt infection. It often changes in parallel with leukocytospermia, elevated sORP, and SDF. In seminal plasma from mixed male-infertility cohorts, *IL-6* and *IL-8* levels are generally higher, and both are associated with poorer semen parameters or testicular dysfunction (55, 93).

*IL-8* (*CXCL8*) is associated with neutrophil recruitment and reduced sperm motility. Proinflammatory signals such as *TNF-α* and *IL-1β* often appear alongside immune cell activation and increased ROS burden. They may collectively contribute to redox imbalance (91). Inflammation and oxidative stress often amplify each other. ROS can trigger proinflammatory signaling, which in turn increases oxidative burden through further leukocyte recruitment (9, 100).

Leukocytospermia is the most direct IO marker in this process, typically co-occurring with cytokine elevation and heightened oxidative load (78, 101). Excess ROS can induce lipid peroxidation in the sperm membrane and may contribute to DNA fragmentation (49). Even when leukocytospermia does not reach the diagnostic threshold, seminal cytokines may still be elevated and remain associated with SDF readouts (102).

Leukocytospermia, cytokine elevation, and oxidative stress therefore converge as related routes linked to increased SDF (92, 103, 104). A meta-analysis reports elevated seminal IL-6 and TNF-α in infertile men, consistent with a mechanistic link to DNA fragmentation via oxidative stress (93).

The strength of Pathway A varies across SD ecotypes. Granulocyte activation markers help identify this subgroup even when leukocyte counts remain below threshold (94). Pathway A deserves priority assessment in anaerobe-enriched or high-load communities (92). In *Lactobacillus*-dominant communities, this chain appears less active and may instead reflect individual variation in host immunity (91).

### 4.3 Mechanistic pathway B: noninflammatory redox imbalance (leukocyte-independent)

The association between SD and ROS/ORP remains largely supported by correlation evidence. Pathway B is shown in **Figure 1**. Endotoxin has been associated with sperm damage through leukocyte-independent mechanisms. Lipopolysaccharide (LPS) and peptidoglycan have been detected in human semen (101). Sperm cells also express toll-like receptor 4 (TLR4). LPS can activate TLR4, which may reduce sperm motility (with partial support from animal models). Even without leukocyte participation, TLR4 activation has been associated with increased sperm apoptosis (105–107).

In infection-positive cohorts (outside the IMI scope defined here), culture positivity is associated with higher semen ROS (108). Dysbiotic communities may reduce seminal antioxidant capacity and increase oxidative-reduction potential, which is associated with sperm motility and DNA integrity (6). Mild ROS increases induced by SD may not suffice to link it to SDF. Damage likely occurs after ROS exceeds the buffering capacity of seminal antioxidants (109). Physiological low-level ROS participates in sperm capacitation. However, excessive ROS become destructive. It has been associated with lipid peroxidation and DNA strand breaks and may impair fertilization and pregnancy outcomes (6, 49).

Some studies report lower sORP in men with leukocytospermia than in those without it (110). These findings suggest that Pathway B operates relatively independently of leukocyte-mediated mechanisms. Abstinence time and semen processing time should also be considered, as shown in **Table 2**. Marker selection should also account for these biological implications (see Section 2.2).

ROS can induce single-strand and double-strand DNA breaks in sperm. The resulting DNA fragmentation is associated with male infertility and adverse reproductive outcomes (49). A cross-sectional study reported that an sORP threshold of about 1.40 mV/10^6^ sperm/mL (AUC = 0.857) could distinguish infertile from fertile men, and this threshold held in samples with or without leukocytospermia (110). This suggests that the ROS–SDF association may not depend entirely on leukocytes. In infertile cohorts, SDF correlates with standardized sORP and is associated with sperm concentration, morphology, and motility (111). When DFI exceeds a critical threshold or oocyte quality declines, the oocyte’s ability to repair paternal DNA damage may fail, contributing to pregnancy loss (97, 112).

### 4.4 Hypothesized route C: metabolic and epigenetic

Compared with Pathways A and B, Route C rests mainly on metagenomic and metabolomic correlations rather than direct causal evidence in human sperm; it is presented here as a hypothesis extrapolated in part from non-reproductive systems (**Figure 1**).

SAM is the sole methyl donor for methyltransferases, supporting DNA methylation and polyamine synthesis (113). Commensal bacteria such as *Bifidobacterium* and *Lactobacillus* can convert one-carbon compounds into SAM (114–117). Metagenomic data from seminal samples of infertile men show marked changes in SAM-cycle, alongside lower sperm concentration (61); whether this reflects upstream metabolic dysregulation during spermatogenesis or post-testicular events remains unresolved. Chromatin remodeling during spermatogenesis depends on such one-carbon substrates, of which SAM availability may be one relevant factor (53, 113, 118).

*Lactobacillus iners* is enriched in men with high DFI, and butyrate has been identified as a candidate target metabolite (62, 119). Insufficient one-carbon metabolism can reduce methyl-donor availability and limit polyamine synthesis. In human spermatogenic cell models, H4K5ac regulates nucleosome disassembly and protamine incorporation, whereas H4K5bu may delay BRDT-dependent nucleosome disassembly; whether microbial butyrate production exerts analogous effects on human sperm chromatin maturation is untested (120).

The weakly alkaline environment of seminal plasma is partly maintained by polyamines, and one-carbon metabolism regulates their synthesis (121, 122). SAM also participates in polyamine synthesis and lipid biosynthesis. SD-associated SAM depletion may affect polyamine homeostasis and the redox buffering capacity of seminal plasma (53, 113). In the seminal metabolomic profiles of men with iOAT, pathways related to inflammation and oxidative stress are enriched in parallel (17, 63, 84).

Defective chromatin packaging caused by impaired protamination can be detected by SCSA/AO, but TUNEL may underestimate it (123, 124). Secondary ROS and inflammation may then more easily cause SSBs and DSBs on this vulnerable substrate (125).

### 4.5 Feedback loops and modifying factors

Dysbiosis-associated microbial communities can be observed in high-viscosity phenotypes. Increased viscosity delays semen liquefaction, which may create a more favorable niche for some genera. Bacteria can adhere to the sperm surface and form aggregates in semen or within ducts. If their ROS exceed the antioxidant buffering capacity of seminal plasma, they can induce membrane lipid peroxidation and leakage of intracellular contents. The released nutrients may then promote further microbial expansion, creating a potential self-reinforcing loop, as shown in **Figure 1** (9).

Some genera, such as *Megasphaera*, are negatively associated with semen viscosity. They may act through acid production or hydrolytic enzymes that degrade seminal proteins (10). Cross-colonization between sexual partners is the hardest modifying factor to control. Among sexually active partners, 56% of dominant genera are shared. In couples with known causes of infertility (for context only), the semen and vaginal microbiomes also show cross-enrichment of *Staphylococcus* and *Streptococcus* (17). Abstinence time is also an important factor. Samples collected after 2 days of abstinence have higher bacterial load and diversity than samples collected after 2 hours of abstinence (57). Longer abstinence is further associated with higher DFI and reduced motility, though no seminal microbiome data currently cover this longer range (58).

The effects of BMI and metabolic status are currently supported mainly by indirect evidence. In a cohort of obese infertile men, the abundance of *Corynebacterium*, *Hydrogenophaga*, and other genera was associated with BMI, and *Brevibacterium* and *Paenibacillus* were associated with SDF. However, the causal chain linking obesity, the seminal microbiome, and SDF has not been established (126).

Probiotics, synbiotics, and antibiotics are the main modulatory approaches with clinical trial data. Their systematic evaluation is presented in Section 5.2. For IMI-core analyses, varicocele should be excluded or reported separately; leukocytospermia and sORP stratification may be necessary to reveal associations between specific SD ecotypes and IO/SDF outcomes.

### 4.6 Minimum testable version

Sections 4.1–4.5 show that SD, IO, and SDF each allow multiple operational definitions. This section proposes a minimum specification for stratification. The testing order is designed for reproducibility and effect enrichment, not to imply causal direction.

#### 4.6.1 Candidate stratification layer

This three-category ecotype classification is a research stratification rather than a validated diagnostic entity. It applies only after decontamination and quality control are completed (Section 2.2), and after restricting sample collection to a defined abstinence window (2–7 days), as abstinence duration confounds bacterial load in this range. Classification proceeds in a fixed order: absolute bacterial load is assessed first to exclude signals within the contamination range, relative-abundance dominance then defines the three ecotypes, and species-level identification is applied as a refinement layer wherever genus-level composition is known to mask divergent functional profiles (40, 41, 90).

(i) *Lactobacillus*-dominant type: Relative Lactobacillus abundance exceeds a locally defined reference threshold, with absolute abundance confirmed by qPCR or flow cytometry. Species-level identification is required at this step (shotgun metagenomics or L. iners-specific primers), as genus-level Lactobacillus dominance does not uniformly predict a favorable redox state; L. iners has been associated with high DFI (62, 127).

(ii) Anaerobe-enriched type: This category does not meet criterion (i). Anaerobic or facultative genera such as *Prevotella* and *Atopobium* (single genus or in combination) reach a prespecified threshold and dominate the community composition, with absolute load above the contamination-range cutoff established previously. Species-level distinctions within these genera carry functional consequences in the vaginal microbiome literature (128), but equivalent seminal species-level data are currently unavailable; this refinement remains exploratory pending semen-specific validation.

(iii) High-load mixed type: Does not meet criteria (i) or (ii); total bacterial load exceeds the reference threshold, no single genus reaches dominance, and α-diversity is usually increased. Species-level resolution is not applicable at this step because no genus reaches the dominance threshold required for targeted identification.

#### 4.6.2 Intermediate phenotype layer

IO is operationalized as two independent binary variables—inflammatory IO and oxidative IO—corresponding respectively to Pathway A and Pathway B. The two are assessed separately, and stratification should specify the resulting combination (inflammatory+/oxidative−, inflammatory−/oxidative+, both, or neither).

Inflammatory IO: Leukocytospermia (yes/no; WHO 6th edition ≥1 × 10^6^ peroxidase-positive cells/mL) serves as the primary screen, with elevated seminal PMN-elastase (ELISA; reference cutoff ≥230 µg/L, local validation required) as a supplementary indicator when leukocyte counts fall below threshold but granulocyte activation is suspected (94).

Oxidative IO: sORP (high/normal, based on the local reference interval; do not apply a single cutoff across studies).

Both markers can be measured routinely in most reproductive medicine laboratories. PMN-elastase requires an additional ELISA step beyond leukocyte counting, so leukocytospermia can substitute for it within the inflammatory IO when resources are limited; however, neither marker is interchangeable with oxidative IO (sORP), which reflects a distinct biological process.

#### 4.6.3 Outcome layer

The primary endpoint should be limited to a single assay platform (e.g., TUNEL or DFI), with the acidic or alkaline protocol, fresh or frozen-thawed sample status, and quality-control procedures specified. Other platforms (SCD, Comet, etc.) should be reserved for sensitivity analyses rather than the primary hypothesis test.

These minimum specifications are methodological trade-offs made to improve reproducibility. The three-category, binary, and single-platform choices each sacrifice some information, but together they yield a falsifiable design. Future studies that use this minimum specification and report negative results will offer a meaningful challenge to the axis. This minimum specification connects with **Table 5**: stratified enrollment depends on the SD and IO binary classification in this section, and methodological reporting follows the STORMS checklist (51). Recommended item: report seminal plasma biochemistry alongside these measures for niche characterization, but do not use it as a hard requirement for SD/IO stratification (15).

**Table 5:** Minimum requirements for future RCT design: the SD–IO–SDF axis.

Design domain	Minimal requirement
Eligibility & stratification (3, 12, 22, 71)	IMI with abnormal semen parameters (e.g., iOAT); if unexplained infertility is included, report separately. Must have both IO stress (inflammatory and/or oxidative) and dysbiosis. Report and stratify by ecotype at baseline
Intervention design (52)	Ecotype may inform trial stratification, not routine treatment selection
Core outcomes (129)	Report: semen analysis, clinical pregnancy, pregnancy loss, live birth, gestational age, birthweight
SDF reporting (130)	State: assay type, protocol variant, sample handling, abstinence time, cut-off source
Microbiome reporting (51)	State: negative controls, decontamination steps, complete STORMS checklist
Follow-up duration (15, 131)	Re-test at ≥ 3 months (one spermatogenic cycle); ideally 6 months
Pregnancy outcomes (129)	Always report pregnancy and live birth, not semen parameters alone

## 5 Translational implications & future directions

### 5.1 Research triage and diagnostic implications

As a screening strategy, cost-effective IO/SDF testing should serve as the first step, with high-quality microbiome testing reserved for abnormal cases at the research level.

**Figure 2** presents a three-step sequence. Step 1 follows the current guidelines and excludes known causes using medical history, semen analysis, endocrine testing, and genetic testing. Step 2 focuses on the remaining idiopathic population and prioritizes IO measurements, such as leukocyte counts and sORP. Cytokine profiling can be added when feasible. Cases that meet the quality-control requirements in Section 2.2 proceed to Step 3, research-based microbiome testing. Otherwise, they do not enter microbiome-level analysis. When NGS is not available, IO/SDF measurements can form an independent evaluation path (leukocytospermia, PMN-elastase, sORP, and routine seminal biochemistry), and SD ecotype classification is a research layer overlay, not a prerequisite for IO/SDF evaluation.

**Figure 2:**
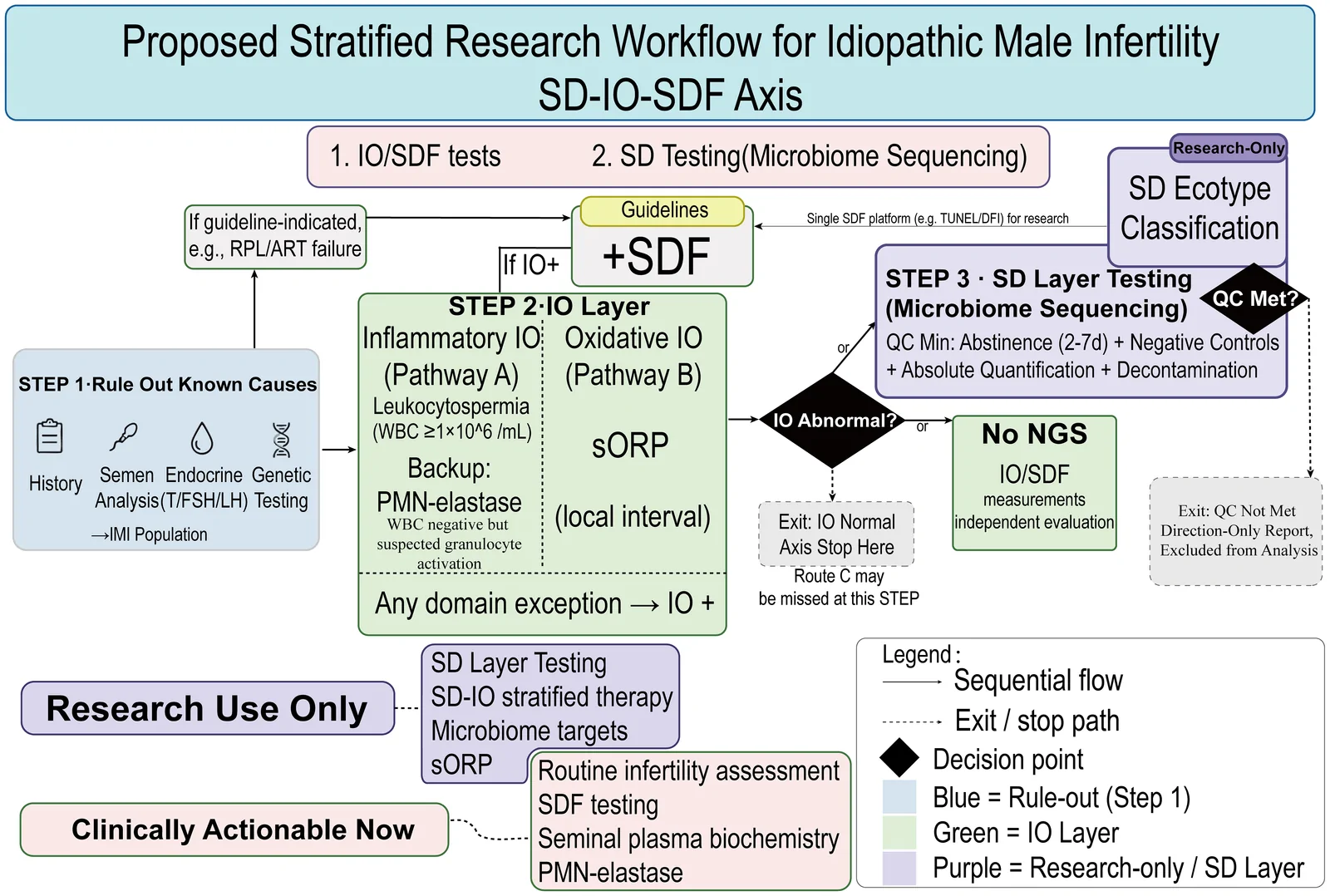
Stratified workflow of the SD–IO–SDF axis. Inflammatory IO (leukocytospermia/PMN-elastase) and oxidative IO (sORP) are not interchangeable. Microbial ecotypes are used only as research-based stratification signals to guide subsequent intervention directions. They are not used as an independent basis for diagnosis or treatment.

Some men may still have dysregulation driven mainly by the mechanisms outlined in Route C (Section 4.4), such as one-carbon metabolism or butyrate signaling. In these cases, IO markers may not yet be abnormal, so they may be missed at Step 2. No data currently show how large this group is. This remains an inherent limitation of the workflow.

Current guideline-concordant uses include routine infertility evaluation, leukocytospermia assessment as part of standard semen analysis, and SDF testing and interpretation in guideline-recommended settings. sORP and other oxidative stress markers are not yet part of routine guideline-endorsed testing.

By contrast, sequencing the ejaculated semen microbiome to define SD ecotypes, SD–IO-based stratified treatment, and using microbiome shifts as treatment targets or surrogate endpoints all remain research-only at this stage. Seminal plasma biochemistry, such as fructose, zinc, NAG, and GPC, can serve as ancillary covariates describing the microbiome’s substrate niche. These markers may help explain differences in compartmental origin (75).

DEFB119 elevation in men with abnormal semen parameters is associated with reduced microbiome diversity and changes in dominant genera. Whether DEFB119 elevation and dysbiosis jointly contribute to semen abnormalities, however, remains to be validated in larger cohorts (132). It has no clinically validated cutoff across studies and should be distinguished from markers with established operational cutoffs, such as sORP and leukocyte counts.

sORP thresholds require laboratory-specific reference intervals rather than published cutoffs (Section 2.2); in Step 2 of **Figure 2**, “abnormal IO” should be defined against local reference populations.

### 5.2 Interventions and management

#### 5.2.1 Systematic evaluation of intervention evidence

Current RCTs fall into three categories: antibiotics, probiotics/synbiotics, and antioxidant combination regimens. Representative studies are listed in **Table 4**, and none of these trials measured the seminal microbiome. Evidence is most abundant for probiotics, but the endpoints almost always remain semen parameters and oxidative stress markers. Antibiotic evidence mainly comes from infection-positive populations and has limited coverage of idiopathic SD populations. The only antioxidant study that reported both sORP and ART outcomes used a nonrandomized design. Existing randomized trials suggest that probiotics or synbiotics can improve semen parameters and oxidative stress markers. However, among the five representative studies in **Table 4**, only one reported improved pregnancy or implantation outcomes, and none showed a significant effect on live birth rates (52).

#### 5.2.2 Why current RCT results are unstable

The unstable effects seen in current RCTs are more likely due to averaging caused by missing stratification than to sample size alone. When IO status is not measured, individuals with activated and nonactivated inflammation are pooled together, and the intervention effect is easily diluted. Different SD ecotypes may also respond differently to probiotics, antioxidants, or antibiotics. In addition, differences in assay platforms and endpoint settings further increase heterogeneity (52, 133–135). Pregnancy and live birth have rarely served as primary endpoints in existing trials, and SDF outcomes needed to test the IO–SDF link are often not fully measured. As a result, current trials make it difficult to determine which part of the SD–IO–SDF axis an intervention actually changes.

#### 5.2.3 Key elements for future RCT design

To address the gaps identified in Section 5.2.2, future trials should complete IO stratification and SD ecotype classification before enrollment. They should pre-specify ecotype-matched intervention arms and include IO, SDF, and pregnancy outcomes among the prespecified endpoints. Methodological reporting should follow current standards, detailed in the checklist in **Table 5**.

### 5.3 Research gaps and priority roadmap

The four gaps below share a common root: all SD–IO–SDF associations reviewed here are classified as preliminary evidence, with unresolved causal direction—and this is precisely where the research opportunities lie.

#### 5.3.1 Causal inference gap

Priority should be given to longitudinal cohorts and repeated measurements before and after intervention, with couple-matched designs included. Follow-up should cover at least one spermatogenic cycle to distinguish “bacteria selected by the seminal environment” from “damage caused by bacteria” (9, 136).

#### 5.3.2 Methodological gap

The low-biomass quality-control and decontamination thresholds listed in Section 2.2, together with the STORMS reporting set, should be treated as mandatory requirements. In addition to relative abundance, absolute quantification or microbial load should be reported. When negative controls are missing, computational contaminant identification can only serve as a fallback measure (31, 51).

#### 5.3.3 Mechanistic validation gap

The field should move from “associated genera” to “candidate molecules.” For targets pointing to butyrate-related pathways in HDFI, *in vitro* exposure and reversibility tests should be conducted, with sperm function and DFI readouts as endpoints (62, 137). The specific contribution of *IL-6* to sperm dysfunction and oxidative stress has not been fully clarified. This suggests that an intermediate step between cytokine activation and functional endpoints still needs to be quantified (138).

#### 5.3.4 Translational gap

The minimum design should link stratification (SD×IO), intervention, and outcomes. The primary endpoint should report IO and SDF together, with repeated microbiome testing. Pregnancy and live birth should be prespecified secondary endpoints. The SDF platform and repeat-testing interval should be standardized according to current research and reporting standards (9, 125).

## 6 Conclusions

The term idiopathic male infertility has limited explanatory power (2–4). The SD–IO–SDF axis proposed here offers a way to stratify this condition (12). By linking seminal dysbiosis, inflammatory/oxidative endophenotypes, and sperm DNA fragmentation, it turns the exclusion-based diagnosis of “idiopathic” infertility into three testable variables.

The three mechanisms are Pathway A (leukocyte-dependent inflammatory–oxidative damage), Pathway B (leukocyte-independent redox imbalance), and Route C (the metabolic–epigenetic mechanism involving the SAM cycle and butyrate). Their relative importance differs across IMI subtypes (17, 63, 84). The evidence for HDFI populations (hypothesis-generating) points more toward the Lactobacillus iners–butyrate link within Route C (62).

In research workflows, measuring IO/SDF first and performing high-quality microbiome testing in abnormal cases may help reduce sequencing noise and enrich testable subgroups (12, 22). It also provides a concrete checklist for future intervention trial enrollment and endpoint design (**Table 5**). At present, this axis is mainly hypothesis-generating. Its clinical value will depend on whether studies stratified by SD and IO can test the contribution of each mechanism of this axis (51, 129).
